# Genome-Wide DNA Methylation Pattern in Whole Blood Associated With Primary Intracerebral Hemorrhage

**DOI:** 10.3389/fimmu.2021.702244

**Published:** 2021-08-13

**Authors:** Yupeng Zhang, Hongyu Long, Sai Wang, Wenbiao Xiao, Meishan Xiong, Jianyi Liu, Lei Chen, Ruijuan Chen, Xueli Wei, Yi Shu, Yi Zeng, Le Zhang

**Affiliations:** ^1^Department of Neurology, Xiangya Hospital, Central South University, Changsha, China; ^2^Department of Geriatrics, Second Xiangya Hospital, Central South University, Changsha, China; ^3^Department of Neurology, Second Xiangya Hospital, Central South University, Changsha, China

**Keywords:** intracerebral hemorrhage, epigenome-wide association study, epigenetics, DNA methylation, stroke

## Abstract

Primary intracerebral hemorrhage (ICH) is a significant cause of morbidity and mortality throughout the world. ICH is a multifactorial disease that emerges from interactions among multiple genetic and environmental factors. DNA methylation plays an important role in the etiology of complex traits and diseases. We used the Illumina Infinium Human Methylation 850k BeadChip to detect changes in DNA methylation in peripheral blood samples from patients with ICH and healthy controls to explore DNA methylation patterns in ICH. Here, we compared genomic DNA methylation patterns in whole blood from ICH patients (n = 30) and controls (n = 34). The ICH and control groups showed significantly different DNA methylation patterns at 1530 sites (p-value < 5.92E-08), with 1377 hypermethylated sites and 153 hypomethylated sites in ICH patients compared to the methylation status in healthy controls. A total of 371 hypermethylated sites and 35 hypomethylated sites were in promoters, while 738 hypermethylated sites and 67 hypomethylated sites were in coding regions. Furthermore, the differentially methylated genes between ICH patients and controls were largely related to inflammatory pathways. Abnormalities in the DNA methylation pattern identified in the peripheral blood of ICH patients may play an important role in the development of ICH and warranted further investigation.

## Introduction

ICH is a significant healthcare and financial burden on society and the families of patients. In Western populations, approximately 10% of all strokes are due to ICH (1), whereas 18.8%-55.4% of all strokes are due to ICH in China (2). ICH is a complex disease caused by environmental and genetic factors (3). Several studies have shown changes in gene expression in the brain following ICH (4, 5). However, the mechanism by which ICH causes these changes remains unknown. There has been recent increasing interest in the roles of epigenetic mechanisms and the interaction between the genome and environment in human diseases (6). Studies have confirmed that epigenetic modifications affect the regulation of gene expression and development, and epigenetics is predicted to be an important part of future genetic studies of ICH (7). However, there are few reports on epigenetic mechanisms in ICH.

DNA methylation refers to the formation of a covalent bond between the 5’ carbon atom of the cytosine of a DNA CpG (5’-cytosine guanine-3’) dinucleotide and a methyl group by DNA methyltransferase, forming 5-methylcytosine (8). DNA hypermethylation can directly inhibit transcription or indirectly inhibit gene expression through transcriptional silencing (9). Methylated DNA in the human genome is an epigenetic marker of paramount importance for normal development. Changes in DNA methylation are associated with cardiovascular and cerebrovascular diseases, such as coronary heart disease, atherosclerosis, and cerebral infarction (10–12). Hypertension is the most important risk factor for ICH (13). Recent studies have found that the overall DNA methylation level of the peripheral blood of patients with essential hypertension was lower than that of the peripheral blood of healthy people (14). Furthermore, an epigenetic study showed that the level of global DNA hydroxymethylation in mouse brains was decreased after ICH (15). While DNA methylation has been linked to gene silencing, DNA hydroxymethylation is associated with actively transcribed genes (16). Therefore, we hypothesized that changes in DNA methylation play an important role in the development of ICH. DNA methylation exhibits strong tissue specificity, and studies of methylation patterns in peripheral blood samples from patients with many neurological diseases, such as Down Syndrome (17) and Parkinson’s disease (18), have indicated that peripheral blood biomarkers are easily used in clinical practice. However, whether DNA methylation is changed in ICH injury remains unknown. In this study, we identified altered genome-wide DNA methylation patterns in whole blood from patients with ICH for the first time.

## Materials and Methods

### Subjects

This case-control study recruited consecutive Han Chinese patients with acute ICH diagnosed at the Department of Neurology in Xiangya Hospital in Changsha, between August 2016 and October 2017. The inclusion criteria were as follows: diagnosis of ICH confirmed by computed tomography (CT) and/or magnetic resonance imaging (MRI); typical areas exhibiting ICH, especially hematoma in the basal ganglia; a clear history of hypertension; 18 years of age or older; and signed informed consent. Cases of mixed cerebrovascular disease (postinfarct hemorrhage or infarct after hemorrhage); ICH caused by arteritis, trauma, drug, tumor, cerebrovascular malformation, cerebral amyloid angiopathy, sinus thrombosis or aneurysm; and patients more than 2 weeks after the onset of ICH were excluded (19, 20). Age- and sex-matched healthy control subjects who had a routine physical check-up for the purpose of health maintenance at Xiangya Hospital were enrolled in the study. The control subjects had no symptoms or history of stroke, autoimmune disease, peripheral atherosclerotic disease, coronary artery disease or hematological disease. Finally, 30 patients and 34 controls were enrolled in the study following the procurement of written informed consent from all subjects. All research protocols were approved by the Ethics Committee of Central South University (No.201503225).

Information related to gender, age, body mass index (BMI), systolic blood pressure (SBP), diastolic blood pressure (DBP), fasting blood glucose (FBG), total cholesterol, triglyceride characteristics, high-density lipoprotein cholesterol, low-density lipoprotein cholesterol was recorded on admission. The hematoma volumes on admission to hospital were determined from base-line computed tomography scans (SOMATOM^®^ Definition Flash; Siemens Healthcare, Erlangen, Germany) by the ABC/2 method for all patients. ICH patients and/or their caregivers were contacted and interviewed at 6 months after index ICH by telephone. We defined favorable outcome as achieving and retaining during entire follow-up an mRS ≤ 3.

### Whole Blood DNA Extraction

Whole blood DNA was extracted from the blood samples of patients and control subjects using a commercial kit (Beijing Adly Biological Company, Beijing, China) according to the manufacturer’s instructions. One microliter of DNA was diluted 10 times with distilled water and shaken evenly, and the DNA concentration (µg/µl) was measured using a NanoDrop spectrophotometer; DNA samples with an OD260/280 ratio from 1.7 to 2.0 were used for further experimentations. The integrity of all DNA samples was assessed by agarose gel electrophoresis and ethidium bromide staining. The main band of the sample electrophoresis is clear, not less than 10kb, no obvious degradation.

### BeadChip DNA Methylation Assay

An Illumina Human Methylation 850k BeadChip Kit was used to determine the pattern and extent of DNA methylation in the samples. DNA samples were processed by a series of steps according to the manufacturer’s protocols: DNA denaturation, whole genome amplification, fragmentation, precipitation, resuspension and hybridization. Signals (grayscale) from the final hybridization products were captured with the iScan system (Illumina, Inc.). The extent of methylation was determined by the β value, which was calculated as β= max (signal B, 0)/[max(signal A, 0)+ max (signal B, 0)+ 100]. The β values range from 0–1, indicating completely unmethylated to totally methylated DNA (21).

### Validation of Methylation by Pyrosequencing

After analysis by Methylation 850k BeadChip, 6 CpG loci methylated to different extents (cg08909806, cg26620021, cg20460771, cg24507266, cg05799811, cg26934362) were selected. Then, we selected blood samples from 15 ICH cases and 15 sex- and age-matched control cases for bisulfite conversion of the target region using a PyroMark PCR kit (Qiagen, 59104). Pyrosequencing was performed using primers designed by PyroMark Assay Design 2.0 software (Qiagen, CA, USA). Pyrosequencing was performed using PyroMark Q96/48 ID (Qiagen, CA, USA).

### Data and Bioinformatics Analyses

BeadStudio Methylation Module v3.2 software (Illumina, Inc.) (http://www.illumina.com) was used to analyze differential DNA methylation sites. Differential methylation sites with p-value<5.92 E-08 according to the Bonferroni correction method were screened to reduce the occurrence of class II statistical errors and ensure the accuracy of the research results. The R (https://www.r-project.org/, version 4.1.0) was used to perform all data analysis. Methylation of gene promoters was defined as methylation in the TSS1500 (upstream), TSS200 (upstream), 5’ UTR and first exon regions. Methylation involving CpG island structures was defined as methylation of a CpG island or the N-shore, S-shore, N-shelf and S-shelf regions, with shores occurring 0–2 kb from a CpG island and shelves occurring 2–4 kb from a CpG island. Differentially methylated genes were selected using the DAVID bioinformatics database (http://david.abcc.ncifcrf.gov/home.jsp/). Pathways enriched in differentially methylated genes were determined using the Gene Ontology (GO) (http://geneontology.org/) and KEGG (http://www.genome.jp/kegg/) databases, and GO terms and KEGG pathways with a Bonferroni corrected p-value of less than 0.05 were reported. Used Pearson’s chi-squared test to compare the distribution of hypomethylation and hypermethylation sites with all probes. The relationship between DNA methylation and clinical outcomes/hematoma volumes was analyzed by Pearson correlation analysis. Measured data are expressed as the mean ± SD. Differences in counted data were statistically analyzed with the chi-square test, and differences in measured data were analyzed by Student’s t-test. p-value < 0.05 indicated statistical significance.

## Results

### Characteristics of Cases and Controls

The total sample population of 64 individuals comprised 30 (aged 41–75 years, the mean age is 54.7) unrelated patients diagnosed with ICH and 34 (aged 33–74 years, the mean age is 54.9) age- and sex-matched unrelated controls (**Table 1**).

**Table 1:** Demographic and clinical characteristics of ICH and control group.

	Patients n=30	Controls n=34	*p*
Age	54.90 ± 7.91	54.71 ± 9.65	0.931
Sex (M/F)	19/11	23/11	0.717
SBP/mmHg	159.7 ± 23.82	138.53 ± 23.59	0.000*
DBP/mmHg	93.7 ± 11.98	86.97 ± 12.76	0.036*
hypertension	30/30	16/34	0.000*
Diabetes	1/30	0/34	0.283
Smoking	11/30	10/34	0.537
Drinking	9/30	9/34	0.754
TC/(mmol/L)	4.97 ± 1.16	4.72 ± 0.81	0.396
TG/(mmol/L)	1.96 ± 0.60	2.07 ± 1.31	0.665
LDL-C/(mmo l/L)	3.20 ± 0.90	2.65 ± 0.73	0.021
HDL-C/(mmol/L)	1.11 ± 0.30	1.19 ± 0.26	0.245
Hematoma volumes/ml	16.95 ± 11.88	/	

Data presented as mean± SD or n (%).

SBP, systolic blood pressure; DBP, diastolic blood pressure; TC, total cholesterol; TG, triglycerides; HDL-C, high-density lipoprotein cholesterol; LDL-C, low-density lipoprotein cholesterol.

*P<0.05.

### Overall Changes in Blood Genomic DNA Methylation in Patients With ICH

**Figure 1:**
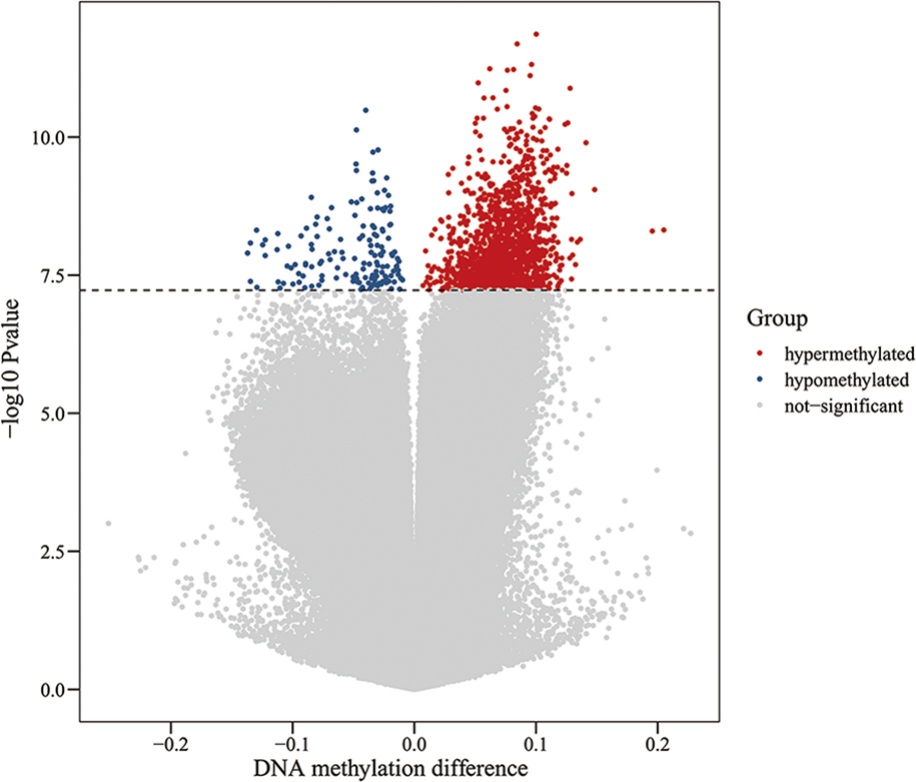
Volcano plot for differential DNA methylation analysis of ICH group and control group. The x-axis shows the DNA methylation difference (delta β), the y-axis shows the – log10 p-value of each CpG site. The dashed line indicates statistically significant (P <5.92 E-08). Red represents hypermethylation sites, blue represents hypomethylation sites, and gray represents no significant.

**Figure 2:**
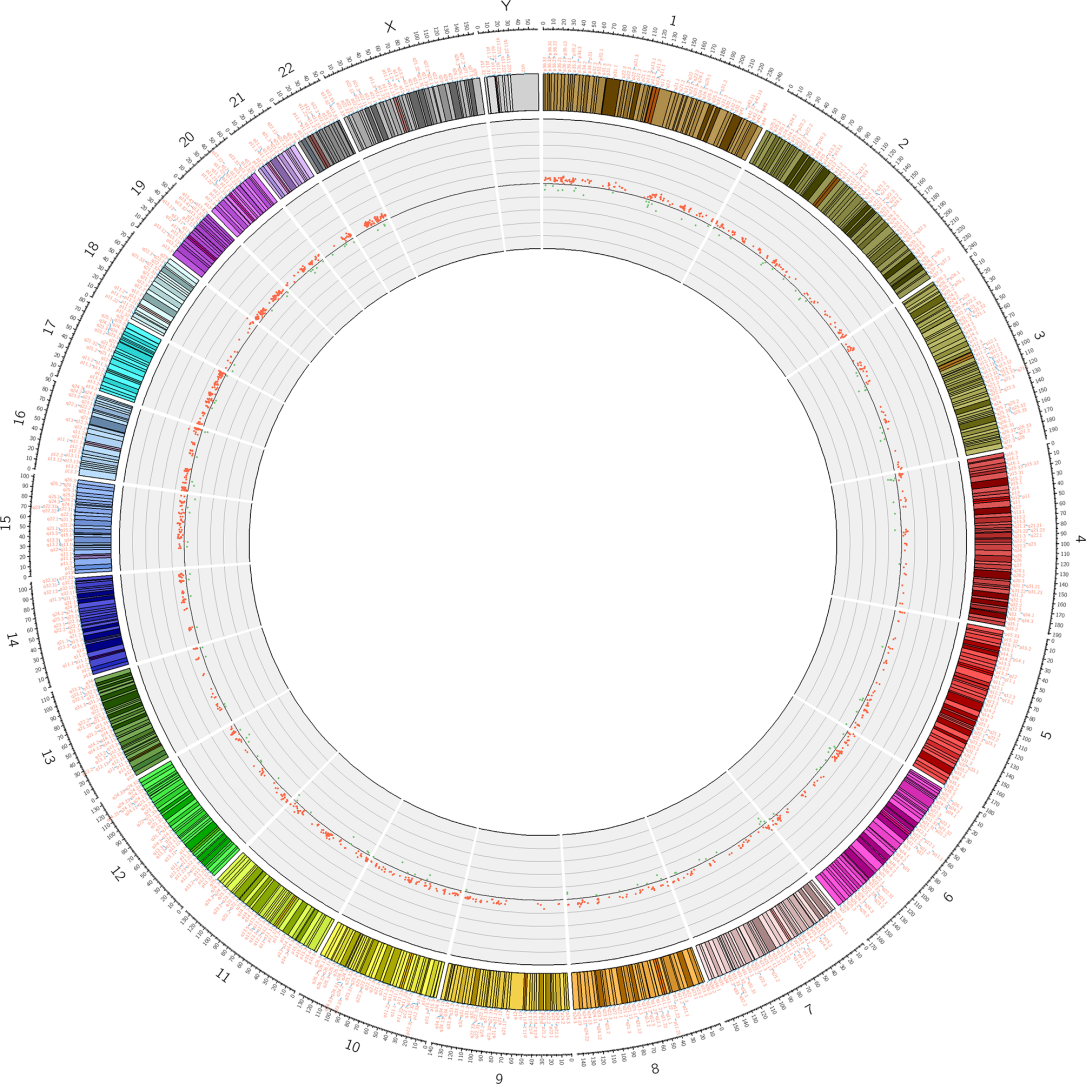
Differential methylation site (P <5.92 E-08) circos diagram. The distribution of 1530 differential DNA methylation sites on 22 chromosomes, the outer layer is the physical location of the chromosome; the middle layer is the chromosome segment; the inner layer is the methylation of the site, red indicates hypermethylation, and green indicates hypomethylation.

**Figure 3:**
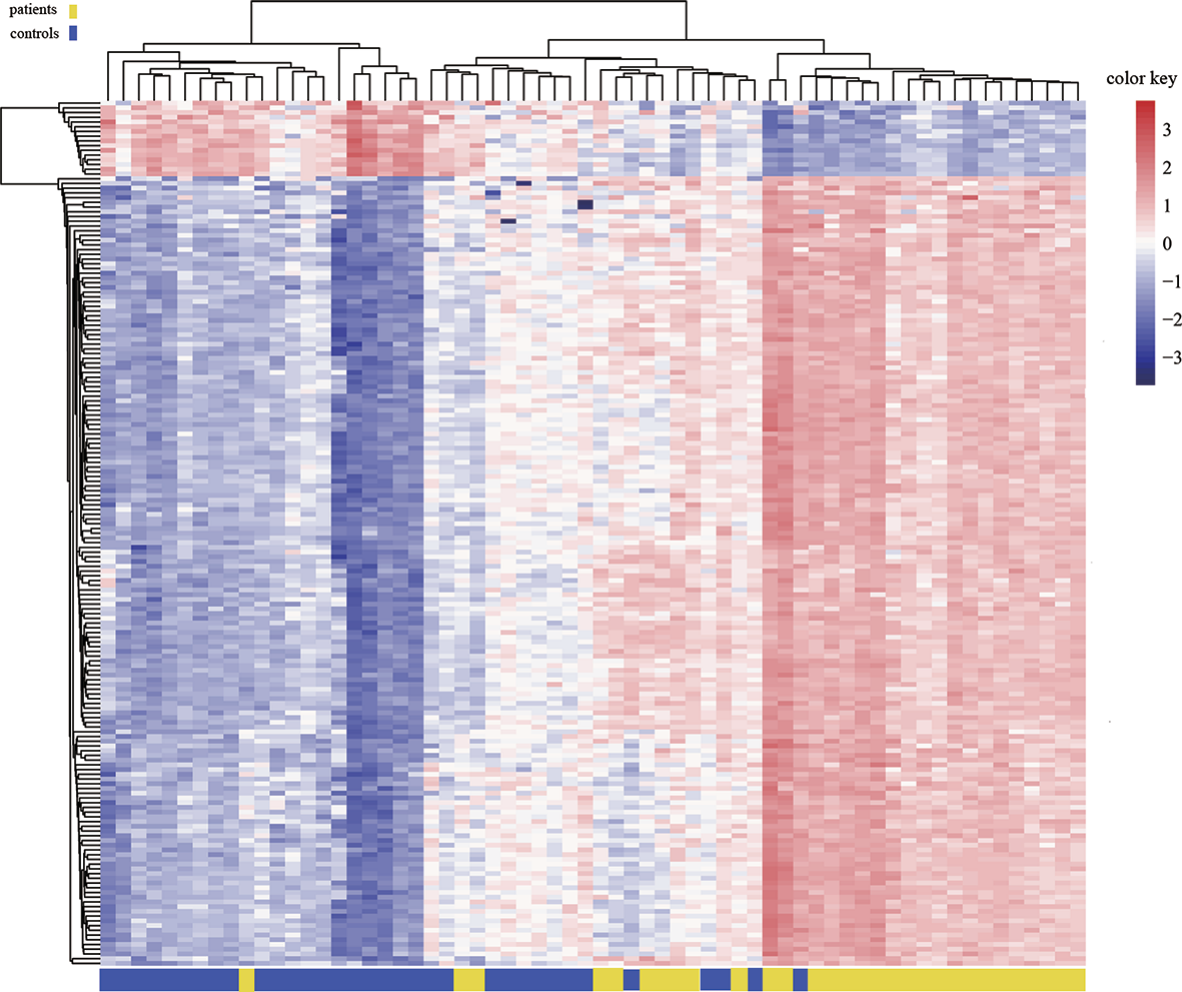
Heat map generated from clustering-analysis of microarray data illustrating differentially methylated DNA sites in blood whole genome in the ICH patient group (n = 30) relative to the control group (n = 34). Blue and red represent levels of hypomethylation and hypermethylation, respectively, whereas white indicates no change in methylation, relative to control.

To assess differences in methylation between the ICH cases and the control group, we conducted genome-wide analysis of DNA methylation in samples from these groups using the Illumina Infinium Human Methylation 850k BeadChip, which allows methylation-specific hybridization to an array of 844668 DNA methylation sites spanning the entire human genome. β-values for sites exhibiting differential DNA methylation between controls (n = 34) and patients with ICH (n= 30) are reported. Statistical analysis showed a total of 1530 loci showing differential DNA methylation between the two groups (defined using cutoff *p*-values < 5.92 E-08) (**Figure 1**). These substantially methylated sites appear to be randomly distributed on 22 chromosomes (**Figure 2**). Of these differential methylation sites, 1377 sites were hypermethylated, and 153 sites were hypomethylated (yielding a ratio of 9) compared to the methylation status of those sites in the control samples (**Figure 3**). The 10 methylation sites with the most significant differences in methylation level are listed in **Table 2**.

**Table 2:** Top ten hyper- and hypo- methylated sites in whole blood genome in ICH.

	TargetID	delta β	*P* value	CHR	GROUP	GENE_NAME
Top ten hypermethylated sites	cg02902412	0.195492157	5.03851E-09	18	Body	*ATP9B*
	cg26934362	0.148252941	8.89275E-10	5	Body	*LPCAT1*
	cg14407179	0.141003922	1.26452E-10	7	Body	*ADAP1*
	cg05799811	0.136335294	7.13005E-09	1	Body	*CD247*
	cg15693299	0.134005882	7.95408E-09	12	TSS1500	*LOC360030*
	cg27642002	0.132466667	2.04617E-08	11	Body	*CLMP*
	cg17972213	0.130166667	1.36499E-08	1	Body	*S1PR1*
	cg13528873	0.129541176	1.0542E-09	6	Body	*GMDS*
	cg20673867	0.129247059	7.13057E-09	9	Body	*MOB3B*
	cg23474890	0.129145098	3.7247E-08	12	Body	*BCL7A*
Top ten hypomethylated sites	cg23351010	-0.1346	4.09969E-08	3	TSS1500;Body	*FLJ10213;PPP4R2*
	cg03330490	-0.13452549	8.20857E-09	10	Body	*HHEX*
	cg08301869	-0.129437255	5.27767E-08	4	Body	*TBC1D14*
	cg11546475	-0.124476471	8.93678E-09	11	Body	*CAT*
	cg07801620	-0.122456863	7.25833E-09	8	1stExon;Body;5’UTR	*PLEC1*
	cg23749353	-0.112090196	5.59553E-09	12	Body	*CORO1C*
	cg05756780	-0.111927451	4.17303E-08	1	Body	*IL6R*
	cg09447621	-0.110345098	5.38782E-08	11	Body	*B3GAT1*
	cg16284789	-0.107533333	4.42562E-08	1	Body	*IL10*
	cg26246740	-0.1034	9.43931E-09	10	TSS1500	*WDFY4*

**Figure 4:**
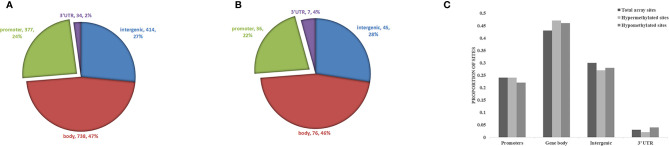
Analysis of differentially methylated DNA sites in reference to genomic structure domains in ICH. **(A)** The distribution of hypermethylation sites, **(B)** the distribution of hypomethylation sites. **(C)** Compared the distribution of hypomethylation and hypermethylation sites with all probes on the Illumina array.

Biological information was available for 1157 sites of the 1530 positive sites, and there were 5 sites that overlap with genetic variant regions (22). Further analysis of DNA functional domains among the hypermethylated sites revealed 414 sites located in noncoding regions (27%), 738 sites in coding regions (47%), 377 sites in promoters (24%) and 34 in the termini of 3’ untranslated regions (UTRs) (2%). Among the hypomethylated sites, 45 sites were in noncoding intergenic regions (28%), 76 sites were in coding regions (46%), 36 sites were in promoters (22%), and 7 (4%) were in the end of a 3’ UTR. There was no significant difference in the distribution of hypomethylation and hypermethylation sites compared with all probes on the 850k Illumina array (**Figure 4**).

**Figure 5:**
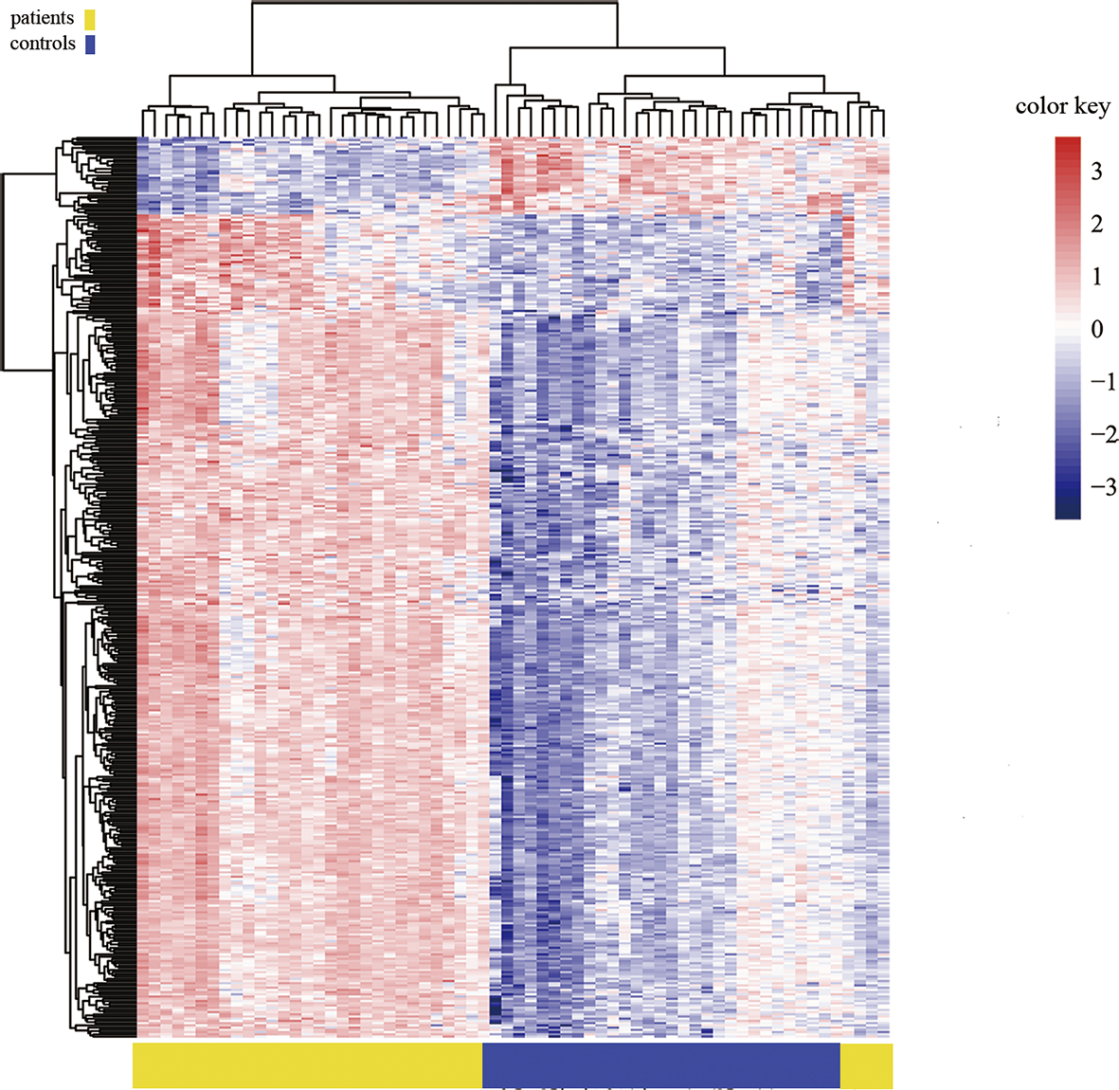
Heat map generated from clustering-analysis of microarray data illustrating differentially methylated DNA sites in the promoter region.

Among the 1530 positive sites, 406 were in promoter regions (**Figure 5**), and among these sites, 371, accounting for 91.38% of the total, were hypermethylated, and 35, accounting for 8.62% of the total, were hypomethylated. Among the 406 methylation sites in promoter regions, 42 were located in a CpG island, 46 were in a north (N)-shore, 59 were in a south (S)-shore, 227 were in an open area, 14 were in a north (N)-shelf, and 18 were in a south (S)-shelf.

### Validation of Methylation by Pyrosequencing

**Figure 6:**
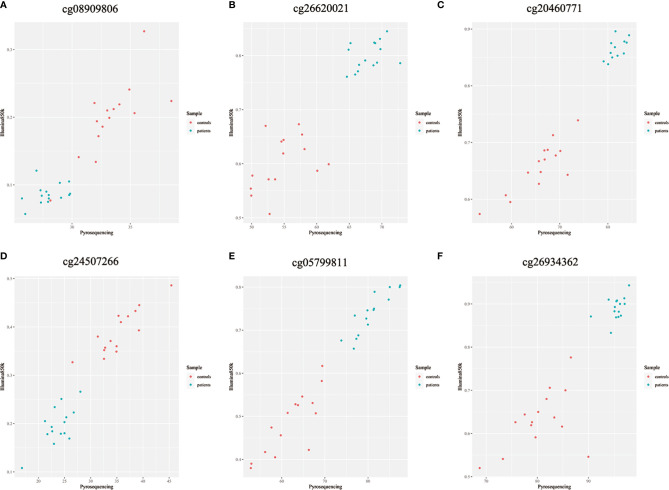
**(A–F)** Methylation chip and pyrosequencing correlation scatter plot Shown are degrees of methylation in 6 CpG loci (cg08909806, cg26620021, cg20460771, cg24507266, cg05799811, cg26934362) reported by Illumina Infinium Human Methylation 850k BeadChip (Y axis, ratio) and pyrosequencing (X axis, percentage) assays. Blue and red dots in each panel plot values of methylation at a given site among individual patients with ICH (n=15) and controls (n=15).

Because previous studies found a good correlation between percent methylation determined by Illumina array and pyrosequencing (23), we randomly selected 6 differential DNA methylation sites for cross-validation with pyrosequencing. Data on both the differential DNA methylation sites and the mean signals for these sites collected by Illumina Infinium Human Methylation 850k BeadChip and pyrosequencing were analyzed. Data on individual sites from Illumina 850k BeadChip array and pyrosequencing were highly correlated, and the means of the differential DNA methylation sites ranged from 0.899 to 0.971 (*p*-value<0.001) (**Figures 6A–F**).

### Ontological Profiling of Differentially Methylated Genes in the Blood of Patients With ICH

**Figure 7:**
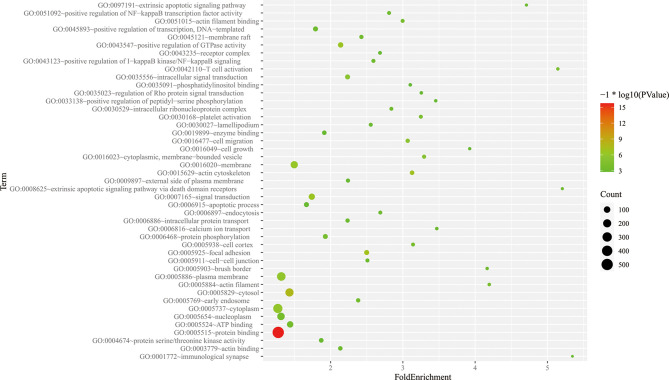
Partial GO pathway enrichment analysis bubble chart. The vertical axis indicates the name of the GO pathway, the horizontal axis is the enrichment factor, and the size of the circle indicates the number of differential methylation-related genes involved in the pathway, The color response of each channel takes p value of – log10.

The 1157 sites with available biological data corresponded to 949 genes. The corresponding biological functions of these genes were obtained from the Database for Annotation, Visualization, and Integrated Discovery (DAVID) database. All genes were enriched in 69 cellular component (CC) terms, and gene enrichment in the following 7 terms was significant (Bonferroni corrected p-value<0.05): (1) GO:0005829, cytosol; (2) GO:0005925, focal adhesion; (3) GO:0015629, actin cytoskeleton; (4) GO:0016020, membrane; (5) GO:0005737, cytoplasm; (6) GO:0005886, plasma membrane; and (7) GO:0016023, cytoplasmic, membrane-bounded vesicle. Among 179 biological process (BP) terms, the following 4 terms were significantly enriched in differentially methylated genes (Bonferroni corrected p-value<0.05): (1) GO:0007165, signal transduction; (2) GO:0043547, positive regulation of GTPase activity; (3) GO:0035556, intracellular signal transduction; and (4) GO:0016477, cell migration. Among the molecular function (MF) terms, 63 terms were enriched in the differentially methylated genes, and 1 of these terms was significantly enriched (Bonferroni corrected *p*-value<0.05): GO:0005515, protein binding (**Figure 7**).

### Genomic Network Profiling of Differential Methylated Genes in the Blood of Patients With ICH

Through bioinformatics assessment against the Kyoto Encyclopedia of Genes and Genomes (KEGG) database (http://www.genome.jp/kegg/), a total of 90 genomic networks/pathways were predicted to exhibit biological interplay between/among differentially methylated genes in ICH, and the 5 following networks/pathways were significant (Bonferroni corrected *p*-value<0.05): (1) hsa04660, T cell receptor signaling pathway; (2) hsa04650, natural killer (NK) cell-mediated cytotoxicity; (3) hsa04662, B cell receptor signaling pathway; (4) hsa04071, sphingolipid signaling pathway; and (5) hsa05205, proteoglycans in cancer (**Table 3**).

**Table 3:** Differential methylation-associated gene KEGG pathway TOP5 in ICH.

Category	Term	Count	Bonferroni *P* value	Genes	Fold Enrichment
KEGG_PATHWAY	hsa04660:T cell receptor signaling pathway	23	6.05E-07	*PTPRC, PTPN6, ITK, TNF, VAV3, CD8A, MAP2K2, NFKBIB, PIK3CD, CD247, RAF1, IL10, NCK2, PRKCQ, MAPK1, FYN, GSK3B, PAK4, ZAP70, PIK3R5, PPP3CA, NFATC2, AKT2*	4.5922908
KEGG_PATHWAY	hsa04650:Natural killer cell mediated cytotoxicity	24	3.33E-06	*PRKCA, PRF1, ITGAL, PTPN6, TNF, VAV3, MAP2K2, CD247, PIK3CD, KLRK1, RAF1, FASLG, GZMB, MAPK1, TNFRSF10D, FYN, ZAP70, KLRC4-KLRK1, PIK3R5, PPP3CA, NFATC2, KLRD1, TYROBP, SH3BP2*	4.045667447
KEGG_PATHWAY	hsa04662:B cell receptor signaling pathway	15	9.76E-04	*PTPN6, VAV3, MAP2K2, NFKBIB, PIK3CD, RAF1, MAPK1, GSK3B, CD81, PIK3AP1, PIK3R5, CD79B, PPP3CA, NFATC2, AKT2*	4.470755694
KEGG_PATHWAY	hsa04071:Sphingolipid signaling pathway	20	9.93E-04	*PRKCA, TNF, SGPP1, MAP2K2, PPP2R5C, PIK3CD, RAF1, MAPK10, SGMS1, ADORA1, TRADD, TNFRSF1A, MAPK1, S1PR1, FYN, S1PR5, PIK3R5, PPP2R5E, PPP2R2C, AKT2*	3.427579365
KEGG_PATHWAY	hsa05205:Proteoglycans in cancer	25	0.007635914	*TNF, CAMK2G, RPS6KB2, FASLG, TGFB1, CTNNB1, IGF1R, CD44, PRKACA, PIK3R5, GPC1, CAMK2A, AKT2, PRKCA, PTPN6, ARHGEF1, MAP2K2, PIK3CD, HSPG2, RAF1, DDX5, ITPR1, MAPK1, SMO, RRAS2*	2.570684524

### The Relationship Between DNA Methylation Patterns and Clinical Outcomes, Hematoma Volumes, Immunological Homeostasis

**Figure 8:**
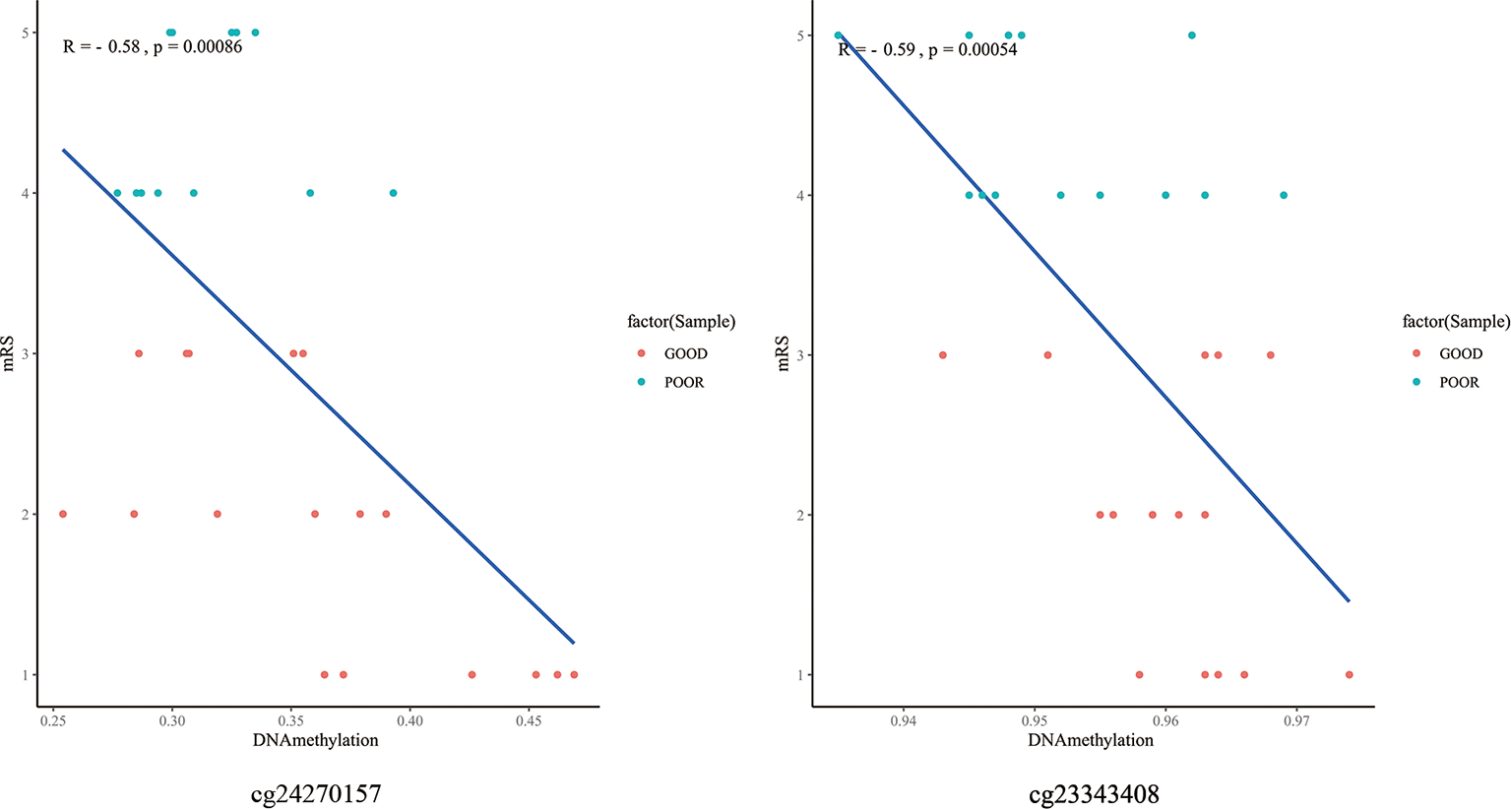
The DNA methylation sites involved in immunological homeostasis and mRS correlation scatter plot. degrees of methylation in cg24270157, cg23343408 are negatively correlated with the 3 months mRS score (R=-0.58, R=-0.59), red dots indicate the favorable outcome patients and the blue means poor outcome.

The top 10 methylation sites correlated with clinical outcomes and hematoma volumes were showed in the **Table 4**. In order to explore the role of immunological homeostasis in the ICH methylation pattern, we screened out the DNA methylation sites in the promoter region related to clinical prognosis, and found the DNA methylation sites involved in immunological homeostasis, including cg24270157(*IL15*), cg23343408 (*TLR5*), which are negatively correlated with the mRS scores (**Figure 8**).

**Table 4:** Top 10 methylation sites correlated with clinical outcomes and hematoma volumes.

	TargetID	*R*	*P* value	CHR	GROUP	GENE_NAME
Top 10 methylation sites correlated with clinical outcomes	cg04128563	0.759939	1.1E-06	6	TSS1500	*AMD1*
	cg21112259	-0.73267	4.2E-06	12	5’UTR;Body	*CSAD*
	cg07624428	-0.72344	6.3E-06	16		
	cg13749939	0.721965	6.7E-06	11	TSS1500	*SLC25A22*
	cg10843426	0.714226	9.3E-06	1		
	cg03770703	-0.71011	1.1E-05	1	TSS1500	*NMNAT2*
	cg27084073	-0.70993	1.1E-05	18		
	cg24764979	-0.70484	1.4E-05	16	TSS1500;5’UTR;1stExon	*GRIN2A*
	cg21669653	0.703948	1.4E-05	12		
	cg13110300	-0.70106	1.6E-05	6		
Top 10 methylation sites correlated with hematoma volumes	cg07877097	-0.80207	9.9E-08	6		
	cg01174235	-0.73588	3.6E-06	7	Body	*WBSCR17*
	cg16909124	0.727268	5.3E-06	12	TSS200	*NR2C1*
	cg20342930	-0.72361	6.2E-06	3	Body	*SPATA16*
	cg08049060	0.720711	7.1E-06	3	TSS200	*TMEM115*
	cg23684711	0.717926	8.0E-06	1	1stExon;5’UTR	*ISG15*
	cg22716633	-0.71382	9.5E-06	4	Body	*PPP2R2C*
	cg00617889	-0.71257	1.0E-05	5		
	cg04662567	-0.71024	1.1E-05	6		
	cg26260819	-0.70911	1.2E-05	11	Body;1stExon	*CRY2*

## Discussion

This is the first report to identify an altered DNA methylation pattern in the whole blood of ICH patients. Using the Illumina Infinium Human Methylation 850k BeadChip, 844668 DNA methylation sites were tested. Differentially methylated sites with a Bonferroni corrected *p*-value < 0.05 were considered significant. We identified 1530 differential DNA methylation sites, among which 1377 sites were significantly hypermethylated, and 153 sites were hypomethylated (yielding a ratio of 9) in ICH patients relative to their methylation in the healthy control group. The methylation of sites around promoters has the strongest inhibitory effect on transcription (24). In this study, among the 1530 positive sites, 406 were located in promoter regions; among these 406 sites, 371 were hypermethylated, and 35 were hypomethylated in patients with ICH, suggesting predominantly increased methylation at these sites. Such changes could enhance the expression of the corresponding genes. This study identified the genome-wide DNA methylation pattern of peripheral blood from patients with ICH and found that the main differences in methylation between ICH patients and normal controls are hypermethylation.

The bioinformatics profile of our data indicates that the differentially methylated regions are correlated to 949 known functional genes. The top ten hypermethylated and top ten hypomethylated genes (**Table 2**), such as *S1PR1* (sphingosine-1-phosphate receptor), the expression of which can improve the clinical outcome of ICH, are related to the pathophysiology of ICH (25). The levels of 5-hydroxymethylcytosine in the CpG-rich regions of the *AKT2*(serine/threonine kinase 2), *PDPK1* (3-phosphoinositide dependent protein kinase 1)and *VEGF* (ascular endothelial growth factor) genes were significantly decreased and at their minimum levels, suggesting the coordinated upregulation of proinflammatory/anti-inflammatory signaling pathways and neuronal signaling systems in ICH mouse brains; furthermore, changes in the expression of these genes contribute to cell death after ICH (15). This confirms the involvement of epigenetic mechanisms such as DNA hydroxymethylation in pathological changes following ICH. Our current data revealed the hypermethylation of the promoters of the *AKT2* and *PDPK1* genes in ICH patients relative to their methylation in the control group. Studies have shown that increased expression of the *TSPO *(translocator protein) gene after ICH may be an intrinsic mechanism that prevents an increase in the inflammatory response (26). The polymorphism rs10940495 in the *IL6ST* (interleukin 6 signal transducer) gene is associated with functional outcome 6 months after ICH (27), transforming growth factor beta 1(*TGF-β1*) modulates microglia-mediated neuroinflammation after ICH and promotes functional recovery (28). The relationship between these genes and ICH needs further research.

KEGG pathway analysis showed that these genes form interconnected networks involved in the T cell receptor signaling pathway, NK cell (natural killer cell)-mediated cytotoxicity, the B cell receptor signaling pathway, the sphingolipid signaling pathway and other networks. The T cell receptor signaling network, which is a proinflammatory signaling pathway, contains three major signaling pathways, the Ca^2+^, mitogen-activated protein kinase (MAPK) kinase and nuclear factor-κB (NF-κB) signaling pathways, that mobilize transcription factors that are critical for gene expression and for T cell growth and differentiation (29). The MAPK family is divided into three subfamilies: p38 MAPKs, extracellular signal-regulated kinases (ERK1/2) and c-Jun amino-terminal kinases (JNKs). Activation of the MAPK pathway protects against ICH-induced secondary brain injury (30). In the present study, genes in the MAPK pathway (*RAF1, MAPK1* and *MAP2K2*) were differentially methylated in ICH patients relative to their methylation status in the control group.

NF-κB signaling pathways plays a key role in immunological homeostasis (31). Previous research indicated a relationship between NF-kB and the pathobiology of perilesional cell death after ICH. In our study, it was found that the methylation pattern of partially DNA methylation sites located in the promoter region was negatively correlated with the follow-up mRS scores of ICH. And these methylation site-associated genes (*IL15, TLR5*) are related to NF-kB signaling pathways (32, 33). We speculate that hypomethylation of the promoter region of these genes may affect their expression and participate in immunological homeostasis of ICH, which is worth-study in the future.

The PI3K/Akt signaling pathway can suppress the Ca^2+^ signaling pathway by glycogen synthase kinase-3β (GSK-3β) in the T cell receptor signaling networks, and statins can increase brain-derived neurotrophic factor (BDNF) and VEGF expression, which activated the PI3K/Akt-mediated signaling signaling pathway and improved neurological function in an autologous ICH model (34). Among the various cytokines that mediate inflammatory reactions in ICH, the proinflammatory cytokines IL, tumor necrosis factor-α (TNF-α) and VEGF play important roles (35–37). In addition, altered methylation in the T cell receptor signaling networks may change the corresponding inflammatory pathways in ICH.

In the present study, genes connected with the NK pathway (*KLRK1, TGFβ1, ITGAL*) were differentially methylated in ICH patients relative to their methylation status in the control group. KLRK1, the receptor for UL16-binding proteins, is expressed on primary NK cells (38), and TGFβ1 expression dramatic reduces surface KLRK1 expression associated with impaired NK cytotoxicity (39). Previous studies suggest that polymorphisms and haplotypes in the *TGFβ1* gene are associated with cerebral infarction and ICH (40, 41). Therefore, far more research on the relationship between changes in the methylation of *KLRK1* and *TGFβ1* with ICH are needed. *ITGAL* gene, which encodes CD11a, is important in adhesive interactions between T cells and other cells of the immune system in the NK pathway. Furthermore, *ITGAL* gene promoter methylation and chromatin structure may contribute to the tissue-specific expression of CD11a (42). However, the relationship between changes in *ITGAL* methylation and ICH is unclear.

There are several limitations to this study. First, we used DNA from peripheral blood leukocytes, representing groups of different cells with different epigenetic profiles. The purpose of this study is to find out some potential epigenetic bio-marks of ICH, future research on more specific types of samples (such as NK-cells, CD8+ T-cells, Neutrophils), which by sorting of subsets by flow cytometry, may provide us with more detailed results. Secondly, the sample size needs to be increased to improve the reliability of the research in the future. Studies have found that primary damage following ICH is mainly the result of the mass effect of hematoma (43). Secondary damage in the brain following ICH occurs through the induction of hematoma toxicity, oxidative stress, cerebral edema and inflammation. Genes associated with the differentially methylated sites identified in this study are primarily involved in inflammatory pathways. DNA methylation is speculated to be involved in the inflammatory response-related mechanism of ICH, which provides new ideas for epigenetic research on ICH. Therefore, further evidence to support proteomics-related studies is still needed.

## Conclusion

We identified 1530 differentially methylated sites between patients with ICH and normal controls for the first time with the Human Methylation 850k BeadChip. Some of the differentially methylated sites identified in the present study are associated with promoters and genes (such as *RAF1*, *MAPK1*, *MAP2K2*, *AKT2*, *KLRK1, TGFβ1, ITGAL*) important for the biological and metabolic regulation of many different inflammatory pathways. Therefore, DNA methylation may play an important role in the pathogenesis of ICH. We hope that future research will combine genomics and proteomics studies to further elucidate the mechanism of DNA methylation in ICH.

## Data Availability Statement

The datasets presented in this study can be found in online repositories. The names of the repository/repositories and accession number(s) can be found below: NCBI GEO GSE179759.

## Ethics Statement

The studies involving human participants were reviewed and approved by Medical Ethics Committee of Xiangya Hospital Central South University (201503225). The patients/participants provided their written informed consent to participate in this study.

## Author Contributions

The co-authors YuZ and HL contributed equally to this article in clinical recruitment,drafted the manuscript and statistical analysis. LZ and YiZ designed the study. SW, MX, JL and LC collected the samples. RC, XW and LW contributed to the reagents, materials, and analysis tools. SW, YS and WX provided technical assistance. All authors contributed to the article and approved the submitted version.

## Funding

This study is funded by the National Science & Technology Foundational Resource Investigation Program of China (Grant No. 2018FY100900), the National Natural Science Foundation of China (Grant No.81571151), the Natural Science Foundation of Hunan Province, China (Grant No. 2016JJ2164) and the Natural Science Foundation of Hunan Province, China (Grant No. 2017JJ2356).

## Conflict of Interest

The authors declare that the research was conducted in the absence of any commercial or financial relationships that could be construed as a potential conflict of interest.

The handling editor declared a shared affiliation with the authors at time of review.

## Publisher’s Note

All claims expressed in this article are solely those of the authors and do not necessarily represent those of their affiliated organizations, or those of the publisher, the editors and the reviewers. Any product that may be evaluated in this article, or claim that may be made by its manufacturer, is not guaranteed or endorsed by the publisher.
